# The prevalence of metabolic syndrome and its association with body fat distribution in middle-aged individuals from Indonesia and the Netherlands: a cross-sectional analysis of two population-based studies

**DOI:** 10.1186/s13098-019-0503-1

**Published:** 2020-01-07

**Authors:** Fathimah S. Sigit, Dicky L. Tahapary, Stella Trompet, Erliyani Sartono, Ko Willems van Dijk, Frits R. Rosendaal, Renée de Mutsert

**Affiliations:** 1 Department of Clinical Epidemiology, Leiden University Medical Center, Albinusdreef 2, 2333 ZA Leiden, The Netherlands; 2 Metabolic, Cardiovascular, and Aging Cluster, The Indonesian Medical Education and Research Institute, Faculty of Medicine-Universitas Indonesia, Jalan Salemba Raya No 6, Jakarta, 10430 Indonesia; 3 Department of Internal Medicine, Dr. Cipto Mangunkusumo National Referral Hospital, Faculty of Medicine-Universitas Indonesia, Jalan Salemba Raya No 6, Jakarta, 10430 Indonesia; 4 Department of Internal Medicine, Leiden University Medical Center, Albinusdreef 2, 2333 ZA Leiden, The Netherlands; 5 Department of Parasitology, Leiden University Medical Center, Albinusdreef 2, 2333 ZA Leiden, The Netherlands; 6 Department of Human Genetics, Leiden University Medical Center, Albinusdreef 2, 2333 ZA Leiden, The Netherlands

**Keywords:** Metabolic syndrome, Abdominal obesity, BMI, Waist circumference

## Abstract

**Background:**

The prevalence of metabolic syndrome varies among populations with different ethnicities. Asian populations develop metabolic complications at lower amounts of adiposity than western populations. The role of abdominal obesity in the metabolic differences between the two populations is poorly understood.

**Objectives:**

Our objectives were to estimate the prevalence of metabolic syndrome and the relative contribution of its components in the Indonesian and the Dutch population, as well as to examine the associations of overall and abdominal obesity with metabolic syndrome.

**Methods:**

In this cross-sectional study of middle-aged adults in the Netherlands Epidemiology of Obesity Study (n = 6602) and the Indonesian National Health Surveillance (n = 10,575), metabolic syndrome was defined by the unified IDF and AHA/NHLBI criteria. We performed logistic and linear regressions to examine associations of BMI and waist circumference with the metabolic syndrome, mutually adjusted for waist circumference and BMI.

**Results:**

The prevalence of metabolic syndrome was 28% and 46% in Indonesian men and women, and 36% and 24% in Dutch men and women. The most prominent components were hypertension (61%) and hyperglycemia (51%) in the Indonesian, and hypertension (62%) and abdominal obesity (40%) in the Dutch population. Per SD in BMI and waist circumference, odds ratios (ORs, 95% CI) of metabolic syndrome were 1.5 (1.3–1.8) and 2.3 (1.9–2.7) in Indonesian men and 1.7 (1.2–2.5) and 2.9 (2.1–4.1) in Dutch men. The ORs of metabolic syndrome were 1.4 (1.2–1.6) and 2.3 (2.0–2.7) in Indonesian women and 1.0 (0.8–1.3) and 4.2 (3.2–5.4) in Dutch women.

**Conclusion:**

More Indonesian women than men have metabolic syndrome, whereas the opposite is true for the Dutch population. In both the Indonesian and the Dutch populations, hypertension is the primary contributor to the prevalence of metabolic syndrome. In both populations, abdominal adiposity was more strongly associated with metabolic syndrome than overall adiposity.

## Background

With the global rise in obesity, metabolic syndrome is becoming a global epidemic as well. It is estimated that 12–37% of the Asian population and 12–26% of the European population suffer from metabolic syndrome [1]. Individuals with metabolic syndrome have an increased risk of cardiovascular morbidity and mortality, as metabolic syndrome is known to be a strong risk factor for type 2 diabetes, cardiovascular diseases, and stroke [2–5].

Metabolic syndrome is defined as a cluster of at least three out of five cardio-metabolic abnormalities which occur concomitantly [2–4]. These abnormalities are abdominal obesity, hyperglycemia, hypertriglyceridemia, low HDL-cholesterol, and hypertension [2–4]. It is unclear to what extent the contributing components differ between populations with a different ethnic background.

Previous studies have demonstrated that cardio-metabolic complications developed at lower amounts of adiposity in Asian than western populations [6]. As an example, a study investigating female populations of Asian-Filipino and Caucasian descents revealed that within a similar range of BMI, the prevalence of type 2 diabetes was higher in Filipinos than in Caucasians (32–36% vs 6–9%) [7, 8]. As a result, recommended cut-offs for BMI and waist circumference are lower in Asian populations, based on their relationship with the increased risks of cardiovascular disease and diabetes mellitus (AHA/NHLBI, IDF, WHO) [2].

One of the explanations for the lower cut-offs in Asians is a relatively higher amount of visceral adipose tissue within the total mass of adiposity, which tends to be lower in Asians than Caucasians. In a study comparing 115 Chinese and 114 white middle-aged men, at the same BMI, Chinese men had more body fat and a higher degree of central fat deposition than white men [9]. Another study examining differences in 1388 Europeans, 838 South Asians, and 330 African-Caribbean showed that South Asians had more and Africans-Caribbean had less visceral adipose tissue than Europeans [10]. Indeed, according to the adipose tissue overflow hypothesis, (South) Asians have a smaller subcutaneous adipose tissue compartment, so their storage capacity is exhausted earlier than Caucasians as obesity develops, and lipids may overflow to the visceral compartment [6]. It is well-established that excess visceral adipose tissue is strongly associated with cardio-metabolic complications [11–18], and may therefore explain the cardiometabolic differences between ethnic populations.

We hypothesized that the differences in abdominal obesity are responsible for the ethnic differences in the prevalence of metabolic syndrome. We aimed to estimate the prevalence of metabolic syndrome, as well as the relative contributions of the components to the metabolic syndrome in the Indonesian and the Dutch population. In addition, we also aimed to investigate the associations of overall and abdominal adiposity with metabolic syndrome, and examine whether the body fat measures associated differently with metabolic syndrome in different ethnic populations.

Several studies have been conducted previously to investigate differences in metabolic syndrome between Asian and Caucasian populations [9, 10, 19–21]. However, they mainly observed Asian populations of Indian and Chinese ancestry, which impedes generalization of the results to the whole Asian population. This study utilized the national database from the Indonesian Health Surveillance, which represents Asian population of Malay-Austronesian origin, who inhabit the majority of geographic area in south-east Asia.

## Materials and methods

### Study design and populations

This study is a cross-sectional analysis of baseline measurements of the Netherlands Epidemiology of Obesity (NEO) study and the 2013 Indonesian Riset Kesehatan Dasar (RISKESDAS) National Health Surveillance.

### The NEO study [22]

The NEO study is a population-based prospective cohort study which includes 6671 individuals aged 45–65 years, with an oversampling of individuals with overweight or obesity. The majority (95%) of the population are White-Caucasian. Between 2008 and 2012, baseline measurements of the NEO study were performed at the Leiden University Medical Center (LUMC), The Netherlands. The Medical Ethical Committee of the LUMC has approved the NEO study, and all participants signed informed consent. Detailed information about the NEO study design and data collection has been described in a previous publication [22].

This present study includes all participants with complete measurements on BMI, waist circumference, blood pressure, as well as blood glucose and lipid profiles (n = 6602).

### The 2013 Indonesian RISKESDAS (National Health Surveillance) [23]

RISKESDAS (Riset Kesehatan Dasar) is a national health survey that is conducted by the Indonesian Government every 5 years. It is designed to represent the Indonesian population nationally to monitor the health status of the citizens, particularly to screen for the presence of infectious, metabolic, and degenerative diseases. A stratified, multi-stage, systemic random sampling design, and the probability proportional to size (PPS) method were used to select households in the 33 provinces across the country. Weighting factors for all individuals have been calculated to ensure that the samples were representative for the different geographical density in the 33 provinces, as well as the urban/rural distribution. The 2013 Indonesian RISKESDAS sampled 1,027,763 respondents for its total study population, including infants and elderly. Among the entire study population, 722,329 respondents were adults aged > 15 years old, of whom 37,891 respondents had been randomly sampled for blood examinations. For the present study, we included adult participants aged 45–65 years who had complete measurements of BMI, waist circumference, blood pressure, and had undergone lipid and glucose blood examination (n = 10,575).

The 2013 Indonesian RISKESDAS methodology has been described comprehensively in previous publications [23]. The present study was approved by and registered in Badan Litbangkes Kemenkes RI (The Indonesian Research & Development Organization, Ministry of Health) [24].

### Data collection

Self-reported questionnaires were used in both studies to assess information on socio-demographic characteristics and risk factors such as age, level of education, smoking behavior, use of sex hormones, frequency and duration of physical activity, and pre-existing cardiovascular disease, stroke, and diabetes.

The Indonesian surveillance provided detailed information on the population’s socio-demographic by categorizing each respondent in urban/rural classification. Socio-economic status in the 2013 Indonesian RISKESDAS was classified by monthly income, asset ownership, and type of housing.

Data on the use of lipid-lowering agents and alcohol consumption were only available in the Dutch population.

### Assessment of overall and abdominal adiposity

In both populations, Body Mass Index (BMI) was used as a proxy of overall adiposity, and waist circumference was used as a proxy for abdominal adiposity. In both populations, waist circumference was measured halfway between the iliac crest and the lowest rib using a flexible steel tape measure to the nearest 0.1 cm. In the Indonesian population, body weight was measured by a calibrated digital FESCO™ weight scale to the nearest 0.1 kg. In the NEO study, body weight was estimated by the Tanita bio-impedance balance (TBF-31-, Tanita International Division, UK) also to the nearest 0.1 kg. Height was measured without shoes using a calibrated, vertically fixed tape measure to the nearest 0.1 cm.

BMI was calculated by dividing body weight (kg) by the square of height (m^2^). Overweight was defined as BMI above ethnic-specific cut-offs, which were ≥ 23 kg/m^2^ for the Indonesian population and ≥ 25 kg/m^2^ for the Dutch population [25].

### Assessment of metabolic syndrome

Metabolic syndrome was defined by the unified IDF and AHA/NHLBI criteria [2], which was at least three out of five cardio-metabolic abnormalities as shown in Table 1.

**Table 1:** The definition of metabolic syndrome [2]

Component	Criteria
Waist circumference above ethnic-specific cut-off	≥ 90 cm in Asian men and ≥ 80 cm in Asian women; ≥ 102 cm in European men and ≥ 88 cm in European women
Elevated triglyceride	≥ 1.7 mmol/L OR use of a lipid-lowering agent(s)
Low HDL-cholesterol	< 1.0 mmol/L in men or < 1.3 mmol/L in women OR use of medication(s) for reduced-HDL
Elevated blood pressure	Systolic BP ≥ 130 and/or diastolic BP ≥ 85 mmHg OR use of anti-hypertensive agent(s)
Elevated fasting glucose	> 5.6 mmol/L OR use of a glucose-lowering agent(s)

The unified IDF and AHA/NHLBI criteria [2]. Metabolic syndrome is defined as at least three out of five cardio-metabolic abnormalities which occur concomitantly

In both populations, blood pressure was obtained by an OMRON™ digital sphygmomanometer at the left arm. In the Dutch population, glucose and lipid (cholesterol and triglyceride) concentrations were determined using standard clinical chemistry methods (Roche Modular P800 Analyzer, Roche Diagnostics, Mannheim, Germany) [22]. In the Indonesian population, lipid profiles were measured with standard clinical chemistry method (autoanalyzer TRX 7010^®^, Tokyo Boeki Medical System, LTD. Japan), whereas glucose profiles were measured with fingertip capillary blood test (Accu-Chek Performa, Roche Diagnostics GmbH, Mannheim, Germany) [23]. All participants had fasted at least 8 h before the blood sampling.

In the Indonesian Health Surveillance, the glucose and lipid blood results were recorded in mg/dL, which we converted to mmol/L for the present study.

### Statistical analysis

Statistical analyses were performed using STATA Statistical Software (StataCorp, College Station, TX, USA), version 14. We adjusted all estimates in both populations for the population-specific sampling design to represent the general Dutch and Indonesian population. To correct for the oversampling of individuals with BMI ≥ 27 kg/m^2^ in the NEO Study, all analyses were weighted towards the BMI distribution of the Dutch general population [22]. To correct for the differences of geographical density across 33 provinces in Indonesia, all analyses in the Indonesian population were weighted towards municipality/provincial density [23].

Results were presented as percentage (SE) for categorical variables, and mean (SD) or median (25th, 75th percentiles) for continuous variables. Results were stratified by sex and ethnicity (Dutch or Indonesian). A 5-variable Venn diagram was composed to illustrate the proportions of the populations with any combinations of components of metabolic syndrome.

Pearson correlation coefficients between BMI and waist circumference were estimated in the Indonesian and Dutch population. We standardized the values of BMI and waist circumference by calculating Z-scores. Logistic regression analyses were performed to examine associations between the standardized measure of BMI and waist circumference with metabolic syndrome and its components: hypertension, hyperglycemia, hypertriglyceridemia, low HDL-cholesterol, except for abdominal obesity. Finally, linear regression analyses were conducted to investigate the strength of the associations between BMI and waist circumference with the components of metabolic syndrome (per mmHg for hypertension, per mmol/L for dyslipidemia and hyperglycemia).

To control for potential confounding, multivariate analyses were adjusted for age, education, smoking status, physical activity, and pre-existing cardiovascular diseases, stroke, and diabetes. Additionally, analyses in the Indonesian population were further adjusted for urban/rural status, as well as the socio-economic position. In the Dutch population, the analyses were additionally adjusted for alcohol intake and the use of sex hormones (in women). Finally, BMI and waist circumference were mutually adjusted to investigate which of the two gave a stronger contribution to metabolic syndrome.

## Results

Table 2 displays the characteristics of the Indonesian and the Dutch populations. Both were adult populations aged 45–65 years, with similarly higher proportions of women than men (60.6% in the Indonesian and 56.2% in the Dutch population).

**Table 2:** Characteristics of the Indonesian and Dutch Cohorts (adult population aged 45–65)

	Indonesian (n = 10,575)		Dutch (n = 6602)	
	Men	Women	Men	Women
n (%)	39.4 (0.6)	60.6 (0.6)	43.8 (0.9)	56.2 (0.9)
Age (years)	53.7 ± 6.5	52.2 ± 5.8	56.0 ± 6.3	55.4 ± 5.8
High education (> 12 years of education) (%)	6.9 (0.6)	4.0 (0.4)	47.9 (1.3)	44.4 (1.3)
Smoking status: (%) current smoker	63.6 (1.1)	4.4 (0.5)	18.3 (1.0)	14.2 (0.9)
Physically active (%)	74.8 (1.1)	80.5 (0.8)	67.7 (1.2)	75.4 (1.1)
Alcohol intake (g/day)^a^	–	–	16.2 (5.7, 28.1)	7.6 (1.5, 14.8)
Menopausal status: (%) postmenopausal	–	–	–	60.2 (1.2)
Urban (%)	46.2 (1.1)	51.8 (0.9)	–	–
Rural (%)	53.8 (1.1)	48.2 (0.9)	–	–
Socio-economic status: (%) highest	20.4 (1.1)	19.0 (1.0)	–	–
BMI (kg/m^2^)	22.6 ± 3.9	24.4 ± 4.6	26.9 ± 3.9	25.9 ± 4.7
Waist circumference (cm)	79.0 ± 11.1	81.8 ± 11.7	98.4 ± 11.4	87.3 ± 12.6
Systolic blood pressure (mmHg)	133.4 ± 22.3	136.4 ± 23.4	134.4 ± 16.1	126.9 ± 16.9
Diastolic blood pressure (mmHg)	82.9 ± 13.0	86.8 ± 12.8	84.8 ± 10.5	81.9 ± 9.9
Fasting plasma glucose (mmol/L)+	6.0 ± 1.8	6.1 ± 2.1	5.7 ± 1.2	5.3 ± 0.8
2-h post-prandial glucose (mmol/L)	8.0 ± 3.2	8.7 ± 3.2	–	–
Triglycerides (mmol/L)	1.6 ± 1.1	1.4 ± 0.8	1.4 ± 1.0	1.1 ± 0.7
HDL cholesterol (mmol/L)	1.2 ± 0.3	1.4 ± 0.3	1.3 ± 0.4	1.7 ± 0.4
LDL Cholesterol (mmol/L)	3.4 ± 0.9	3.6 ± 1.0	3.5 ± 1.0	3.5 ± 0.9
Total cholesterol (mmol/L)	5.0 ± 1.1	5.3 ± 1.1	5.5 ± 1.1	5.8 ± 1.0
Total body fat (%)	–	–	25.0 ± 6.1	36.9 ± 6.4
Fat mass (kg)	–	–	22.6 ± 9.1	27.5 ± 9.9
Lean body mass (kg)	–	–	65.4 ± 7.2	44.8 ± 4.3
Use of anti-hypertensive medication (%)	4.4 (0.4)	7.7 (0.5)	24.2 (1.0)	22.7 (1.0)
Use of oral anti-diabetic medication (%)	2.9 (0.3)	3.5 (0.4)	2.4 (0.2)	1.6 (0.2)
Use of insulin injection (%)	0.4 (0.1)	0.5 (0.2)	0.4 (0.2)	0.3 (0.1)
Use of lipid-lowering agents (%)	–	–	14.9 (0.8)	7.4 (0.6)
Pre-existing cardiovascular disease (%)	1.1 (0.2)	1.4 (0.2)	7.5 (0.7)	4.2 (0.5)
Pre-existing stroke (%)	1.2 (0.2)	1.4 (0.3)	2.0 (0.4)	2.1 (0.4)
Pre-existing diabetes (%)	3.9 (0.4)	4.6 (0.4)	4.3 (0.4)	3.1 (0.3)
Diabetes diagnosis—ADA criteria (fasting glucose)	9.6 (0.6)	11.3 (0.6)	6.1 (0.5)	3.2 (0.3)

Data were presented in mean ± SD or % (SE). Results were based on analyses weighted towards geographical density across 33 provinces (in the Indonesian population) and towards a normal BMI distribution (in the Dutch population)

^a^Indicating a not normal distribution; presented in median (25th, 75th percentiles)

Waist circumference cut-off: ≥ 90 cm in Asian men and ≥ 80 cm in Asian women; ≥ 102 cm in European men and ≥ 88 cm in European women)

BMI overweight cut-off: ≥ 23 kg/m^2^ in Asian and ≥ 25 kg/m^2^ in European population

Impaired Fasting Glucose cut-off: ≥ 5.6 mmol/L

ADA Criteria: Fasting Glucose ≥ 7 mmol/L or 2-h postprandial glucose ≥ 11.1 mmol/L

The mean (SD) age was 53.7 (6.5) and 52.2 (5.8) in Indonesian men and women, whereas in the Dutch population, the mean (SD) age was 56.0 (6.3) in men and 55.4 (5.8) in women. The proportion of participants with high education was higher in the Dutch population (47.9% in men and 44.4% in women) than in the Indonesian (6.9% in men and 4.0% in women). Indonesian men had the highest percentage of smokers (63.6%).

The proportions of overweight according to the ethnic-specific BMI cut-off were highest in Indonesian women (58.2%) and Dutch men (67.0%). The use of antihypertensive medications was substantially higher in the Dutch (24.2% in men, 22.7% in women) than in the Indonesian population (4.4% in men, 7.7% in women).

The prevalence of self-reported diabetes was 3.9% in Indonesian men, 4.6% in Indonesian women, 4.3% in Dutch men, and 3.1% in Dutch women. The proportions of the population with fasting plasma glucose ≥ 7 mmol/L were 9.6%, 11.3%, 6.1%, and 3.2%, respectively.

Correlation coefficients between BMI and waist circumference were 0.75 in the Indonesian and 0.81 in the Dutch population.

### The prevalence of metabolic syndrome and its components

Table 3 presents the prevalence of metabolic syndrome and its components in the Indonesian and Dutch population. The prevalence of metabolic syndrome was 39.0% in the Indonesian and 29.2% in the Dutch population. The sex-stratified prevalence was 28.0% and 46.2% in Indonesian men and women, and 36.2% and 23.8% in Dutch men and women, respectively.

**Table 3:** The prevalence of metabolic syndrome and its components in the Indonesian and Dutch population

	Indonesian (n = 10,575)			Dutch (n = 6602)		
	Total	Men	Women	Total	Men	Women
Metabolic syndrome	39.0 (0.7)	28.0 (0.9)	46.2 (1.0)	29.2 (0.7)	36.2 (1.1)	23.8 (0.9)
Abdominal obesity	41.5 (0.8)	16.8 (0.8)	57.5 (1.0)	40.0 (0.8)	34.6 (1.0)	44.2 (1.2)
Hyperglycemia	51.0 (0.8)	51.0 (1.0)	50.9 (1.1)	30.9 (0.8)	40.5 (1.2)	23.4 (1.0)
Hypertriglyceridemia	27.1 (0.6)	31.6 (1.0)	24.2 (0.8)	25.3 (0.7)	34.9 (1.2)	17.7 (0.8)
Low HDL-C	38.7 (0.7)	28.6 (0.8)	45.3 (0.9)	23.2 (0.7)	27.1 (1.1)	20.1 (0.9)
Hypertension	61.3 (0.8)	56.2 (1.0)	64.6 (1.0)	61.7 (0.9)	70.0 (1.3)	55.2 (1.3)

Data were presented in % (SE). Results were based on analyses weighted towards geographical density across 33 provinces (in the Indonesian population) and towards a normal BMI distribution (in the Dutch population)

Hypertension was the most prominent metabolic syndrome component in both the Indonesian (61.0%) and the Dutch (62.0%) population, followed by hyperglycemia in the Indonesian (51.0%) or abdominal obesity in the Dutch (40.0%) as the second most common contributing component.

In the Indonesian population, women had a higher prevalence of abdominal obesity (57.5% vs. 16.8%), low HDL-cholesterol (45.3% vs. 28.6%), and hypertension (64.6% vs. 56.2%) than men.

In the Dutch population, except for abdominal obesity, men had a higher prevalence of four out of five components of metabolic syndrome than women.

### The contribution of metabolic syndrome components in the population

**Fig. 1:**
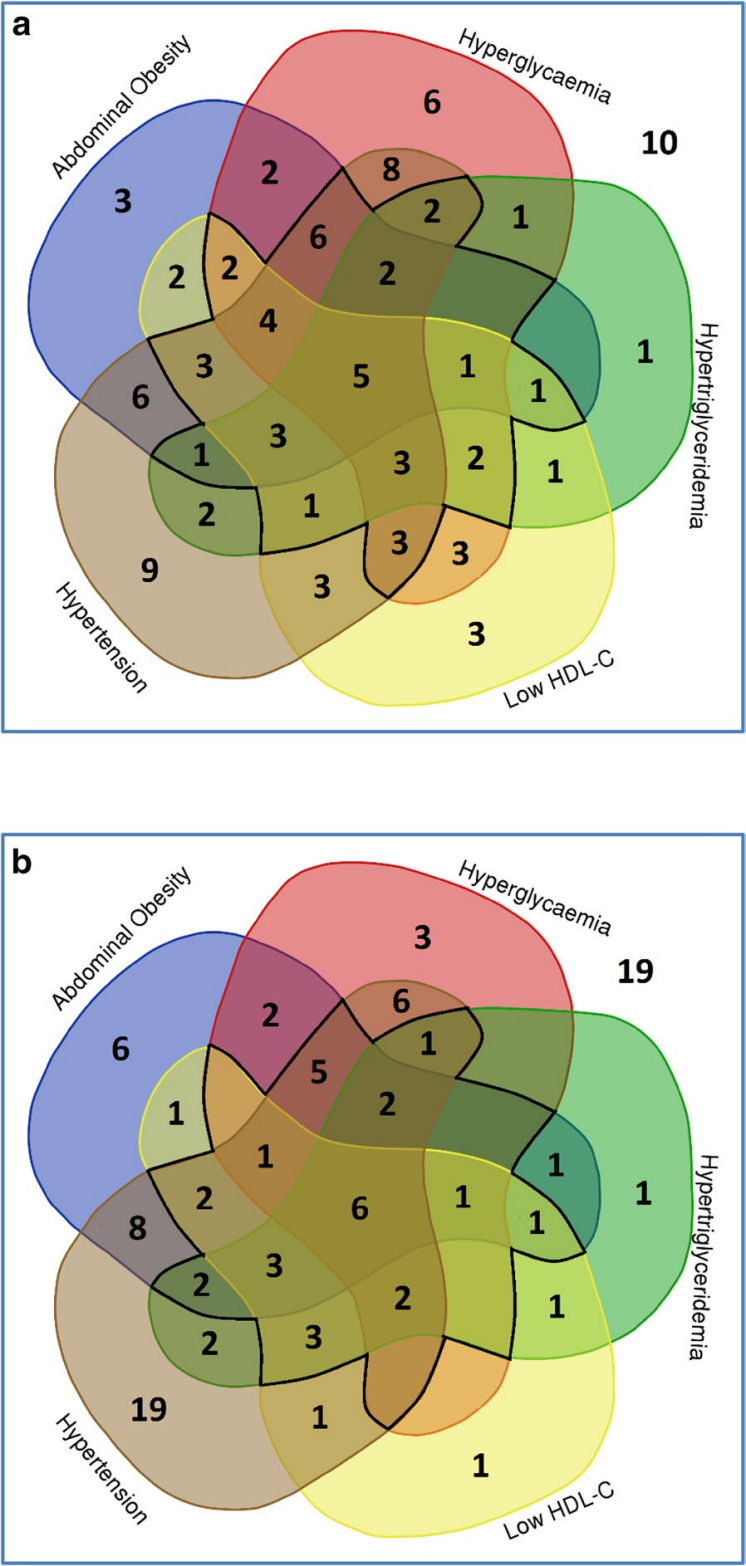
The contributions of metabolic syndrome components in the Indonesian and Dutch population. **a** The contributions of components in the Indonesian population. **b** The contributions of components in the Dutch population. The colorful area within the inner bold black line represents the proportions (%) of the population with metabolic syndrome (having concomitantly 3, 4, or 5 components). A blank area represents proportions of ≤ 0.4%. The number outside the colorful area represents the proportion of the population with no abnormalities

Figure 1 is a 5-variable Venn Diagram that demonstrates the proportions of the population with any combination of components of the metabolic syndrome. The exact proportions of each combination of components in the two populations are shown in Additional file 1: Table S1.

There were 10% and 19% of the Indonesian and Dutch adults who had no cardio-metabolic abnormalities. There were 23% and 30% of the two populations that had one component, and 28% and 21% that had two components of metabolic syndrome.

Among the Indonesian population, the proportion of individuals with metabolic syndrome who possessed three, four, and five components were 22%, 12%, and 5%, respectively. The numbers were 14%, 10%, and 6% among the Dutch population (Additional file 1: Table S1). A subpopulation analysis conducted within metabolic syndrome patients shows hypertension as the most contributing component (Additional file 1: Table S2).

### The associations of overall and abdominal adiposity with metabolic syndrome and its components

Table 4 presents the odds ratios of metabolic syndrome and its components, except for abdominal obesity, per standard deviation (SD) of BMI and waist circumference. The standard deviations were 4.4 kg/m^2^ for BMI in both populations, 11.6 cm for waist circumference in the Indonesian population, and 13.4 cm for waist circumference in the Dutch population.

**Table 4:** The associations of overall and abdominal adiposity with metabolic syndrome and its components

BMI	Indonesian (SD = 4.4 kg/m^2^)				Dutch (SD = 4.4 kg/m^2^)			
	Men		Women		Men		Women	
	Crude OR	Adjusted OR	Crude OR	Adjusted OR	Crude OR	Adjusted OR	Crude OR	Adjusted OR
Metabolic Syndrome	3.0 (2.7, 3.4)	1.5 (1.3, 1.8)	2.3 (2.1, 2.5)	1.4 (1.2, 1.6)	5.0 (4.1, 6.2)	1.7 (1.2, 2.5)	3.4 (3.0, 3.8)	1.0 (0.8, 1.3)
Hypertension	1.6 (1.4, 1.8)	1.3 (1.1, 1.6)	1.5 (1.4, 1.6)	1.4 (1.2, 1.5)	2.0 (1.7, 2.3)	2.0 (1.4, 3.0)	1.6 (1.4, 1.7)	1.5 (1.2, 1.9)
Hypertriglyceridemia	1.7 (1.6, 1.9)	1.3 (1.1, 1.5)	1.4 (1.3, 1.5)	1.3 (1.1, 1.4)	2.3 (2.0, 2.6)	1.4 (1.0, 1.9)	1.9 (1.8, 2.1)	0.9 (0.7, 1.1)
Low HDL-C	1.5 (1.3, 1.7)	1.2 (1.1, 1.4)	1.3 (1.3, 1.4)	1.3 (1.1, 1.4)	2.0 (1.7, 2.3)	1.4 (1.0, 1.8)	2.0 (1.8, 2.1)	0.8 (0.7, 1.0)
Hyperglycemia	1.3 (1.2, 1.4)	1.3 (1.1, 1.4)	1.1 (1.1, 1.2)	1.1 (1.0, 1.3)	2.0 (1.8, 2.3)	1.7 (1.2, 2.4)	2.0 (1.8, 2.2)	1.4 (1.1, 1.8)

Waist circumference	Indonesian (SD = 11.6 cm)				Dutch (SD = 13.4 cm)			
	Men		Women		Men		Women	
	Crude OR	Adjusted OR	Crude OR	Adjusted OR	Crude OR	Adjusted OR	Crude OR	Adjusted OR
Metabolic syndrome	3.1 (2.8, 3.5)	2.3 (1.9, 2.7)	2.8 (2.5, 3.1)	2.3 (2.0, 2.7)	4.9 (4.1, 5.8)	2.9 (2.1, 4.1)	4.6 (4.0, 5.2)	4.2 (3.2, 5.4)
Hypertension	1.6 (1.4, 1.7)	1.3 (1.1, 1.5)	1.5 (1.4, 1.6)	1.2 (1.1, 1.4)	1.7 (1.5, 2.0)	0.9 (0.6, 1.3)	1.5 (1.4, 1.7)	1.0 (0.8, 1.3)
Hypertriglyceridemia	1.7 (1.5, 1.8)	1.3 (1.2, 1.5)	1.5 (1.4, 1.6)	1.2 (1.1, 1.4)	2.2 (2.0, 2.5)	1.4 (1.1, 2.0)	2.3 (2.1, 2.6)	2.3 (1.8, 3.0)
Low HDL-C	1.5 (1.4, 1.6)	1.4 (1.2, 1.6)	1.3 (1.2, 1.4)	1.2 (1.1, 1.3)	1.9 (1.7, 2.2)	1.3 (1.0, 1.8)	2.3 (2.1, 2.6)	2.4 (1.9, 3.0)
Hyperglycemia	1.2 (1.1, 1.3)	1.0 (0.9, 1.1)	1.2 (1.1, 1.2)	1.1 (1.0, 1.2)	1.9 (1.6, 2.1)	1.0 (0.7, 1.4)	2.2 (2.0, 2.5)	1.4 (1.0, 1.9)

**Fig. 2:**
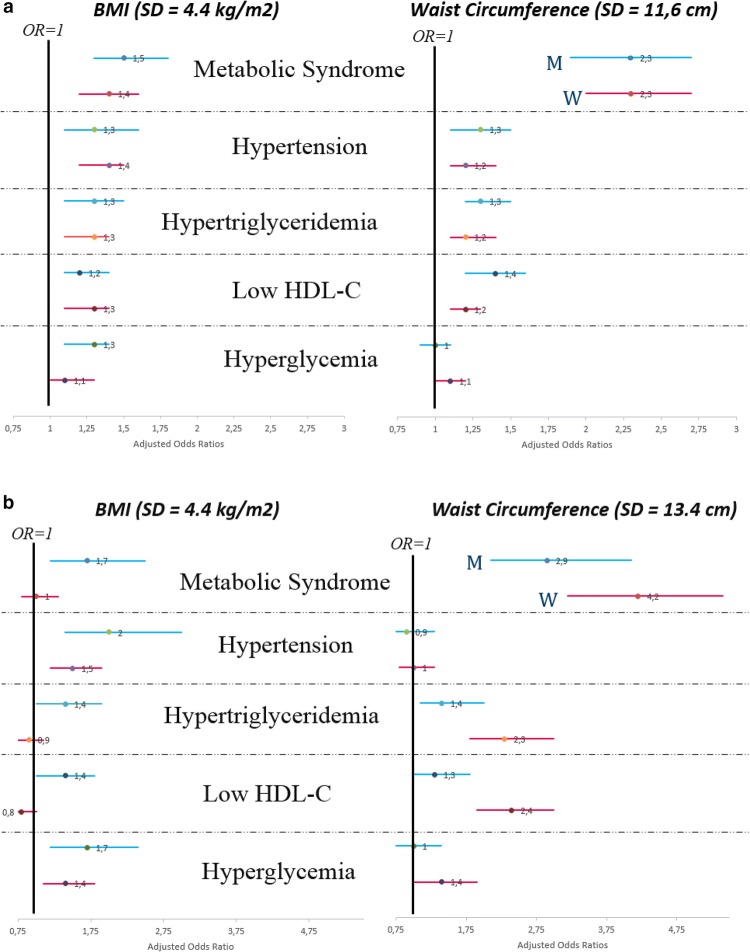
The Associations of Overall and Abdominal Adiposity with metabolic syndrome and its components. The forest plot showed the adjusted Odds Ratios of Metabolic Syndrome and its components per 1 SD of BMI (4.4 kg/m^2^) and per 1 SD of waist circumference (11.6 cm in the Indonesian, 13.4 cm in the Dutch population). Data were presented in OR (95% CI). BMI and waist circumference were mutually adjusted. See Table 4. **a** **The associations in the Indonesian population**. Models were adjusted for age, education, smoking behavior, physical activity, pre-existing CVD, Stroke, and Diabetes, urban/rural, and socioeconomic status. **b** **The associations in the Dutch population**. Models were adjusted for age, education, smoking behavior, physical activity, pre-existing CVD, Stroke, and Diabetes, and alcohol consumption. In women: plus menopausal status and sex hormone use

The Odds Ratios of Metabolic Syndrome and its components per 1 SD of BMI and waist circumference. Data were presented in OR (95% CI). Interpretation: 1 SD of waist circumference in Indonesian men is associated with 2.3× higher risk of metabolic syndrome after adjustment. Multivariate were adjusted for age, education, smoking behavior, physical activity, pre-existing CVD, Stroke, and Diabetes. In the Indonesian population: additionally adjusted for urban/rural, and socioeconomic status. In the Dutch population: additionally adjusted for alcohol consumption, menopausal status, and hormone use. BMI and waist circumference were mutually adjusted. See Fig. 2

Per SD in BMI and waist circumference, the odds ratios of metabolic syndrome were 1.5 (1.3–1.8) and 2.3 (1.9–2.7) in Indonesian men, as well as 1.4 (1.2–1.6) and 2.3 (2.0–2.7) in Indonesian women (Fig. 2a). In the Dutch population, the odds ratios of metabolic syndrome were 1.7 (1.2–2.5) and 2.9 (2.1–4.1) in men, as well as 1.0 (0.8–1.3) and 4.2 (3.2–5.4) in women, respectively (Fig. 2b).

When considering linear associations with the components as continuous outcomes, both overall (BMI) and abdominal (waist circumference) adiposity were similarly associated with higher serum triglyceride, higher fasting plasma glucose, and lower HDL-cholesterol (Additional file 1: Table S3). An exception exists for hypertension, particularly in the Dutch population, as the associations between waist circumference and blood pressure attenuated after adjustment for BMI, whereas the associations of BMI and blood pressure remained stable after adjustment for waist circumference. In Dutch women, the association between BMI with hypertriglyceridemia and low HDL-cholesterol disappeared after adjustment for waist circumference (Table 4 and Additional file 1: Table S3).

## Discussion

The prevalence of metabolic syndrome in adults aged 45–65 was 39.0% in the Indonesian and 29.2% in the Dutch population, which was further detailed as 28.0% and 46.2% in Indonesian men and women, and 36.2% and 23.8% in Dutch men and women. Hypertension was the most prominent metabolic syndrome component in both populations, followed by hyperglycemia (in the Indonesian) and abdominal obesity (in the Dutch) as the second most common contributing component.

Abdominal adiposity, rather than overall adiposity, was more strongly associated with metabolic syndrome. This was most distinctly seen in Dutch women, with a fourfold increased risk per SD of waist circumference. An exception existed for hypertension, being a component that was more BMI-driven, as the association of waist circumference with hypertension disappeared after adjustment for BMI.

The prevalence of metabolic syndrome in this present study was higher than in previous studies conducted among Indonesian and Dutch populations. In 2000, in a study conducted in Jakarta Province (Indonesia) involving 352 women and 137 men aged 55–85, the prevalence of metabolic syndrome (NCEP/ATPIII) was 18.2% and 6.6% [26]. In 2006, still in Jakarta Province, involving 1800 inhabitants aged 25–64, the prevalence of metabolic syndrome (NCEP/ATPIII) was 25.4% in men and 30.4% in women [27]. The higher prevalence in the present study may be explained by the different age of the studied populations: one study included older individuals [26], the other was younger [27]. These two studies were also being exclusive for citizens living in the Jakarta province, whereas we studied an Indonesian nationwide sample [23, 26, 27]. Besides, our study represents more recent data (obtained in 2013) compared with the two previous studies which were conducted in 2000 and 2006, over which time the prevalence may have truly increased. Our results are in line with the growing national burden of non-communicable disease in Indonesia, as between 1990 and 2016 disability-adjusted life years (DALYs) had risen for non-communicable diseases, particularly ischemic heart disease, cerebrovascular disease, and diabetes as the three leading causes of DALYs [28]. This was also supported with previous studies that reported a rapid increase of the prevalence of overweight/obesity and diabetes in Indonesia over the past decades [29, 30].

In the Dutch population, the prevalence of metabolic syndrome in this present study was also higher than previously reported. The Dutch Lifelines Cohort Study, conducted among 74,531 adults aged 18–79 in the Netherlands, reported a prevalence of metabolic syndrome of 19.2% and 12.1% in men and women [31]. Another study involving adults aged 28–59 (from the MORGEN and PREVEND studies) showed that the prevalence of metabolic syndrome was 16–19% in men and 10–12% in women [32]. The lower prevalence of metabolic syndrome in the previous studies may be due to the inclusion of younger, and thus healthier, individuals in these studies, in addition to the higher cut-off of hypertension in the aforementioned studies (≥ 140/90 mmHg for those aged < 60, and ≥ 150/90 mmHg for those aged > 60) [31, 32].

However, the observed sex differences in the prevalence of metabolic syndrome were in line with the previous literature, as all aforementioned studies confirmed that the prevalence of metabolic syndrome was higher in Indonesian women than in Indonesian men, but higher in Dutch men than in Dutch women [26, 27, 31, 32]. The sex-disparities in the prevalence of metabolic syndrome may be explained by the prevalence of abdominal obesity in Indonesian women (57.5%) which was strikingly higher than in men (16.8%), as well as the discrepancy in the prevalence of low HDL-C (45.3% vs 28.6%). In contrast, in the Dutch population, men had almost twofold the prevalence of hypertriglyceridemia and hyperglycemia than women (34.9% compared with 17.7%, and 40.5% compared with 23.4%).

This study confirmed several findings from previous studies conducted among Asian and Caucasian populations. In a study among White-American and Korean-Asian population, Korean Asian had higher prevalence of low HDL-cholesterol than White-American [19]. In another study among Asian-Filipino and Caucasian Women, Filipino women had lower levels of HDL-cholesterol and higher levels of triglycerides, as well as a higher prevalence of T2D (32.6–36.0% vs. 6.1–9.0%) and metabolic syndrome (32.6–34.0% vs. 13.0–13.8%) than Caucasian women [7, 8]. Another study reported that Asian-Chinese men had higher levels of triglycerides and fasting glucose than European men, as well as Chinese women had higher glucose levels than European women [33]. Our study adds to these previous literature as the first to use Asian-Indonesian nationwide population data to support the relevance and generalizability in the wider Asian population.

In both populations, abdominal adiposity was more strongly associated with metabolic syndrome than overall adiposity. The higher odds ratio of metabolic syndrome per waist circumference, rather than per BMI, may partially due to abdominal obesity being also a component of metabolic syndrome. Nevertheless, when considering separate associations with individual components of metabolic syndrome, hypertension appeared to be more BMI-driven, particularly in the Dutch population. The associations of waist circumference and blood pressure attenuated after adjustment for BMI, whereas the associations of BMI and blood pressure remained stable after adjustment for waist circumference. This may be explained by the theory that obesity results in an increase of cardiac output and an expanded intravascular volume, which then lead to left ventricular hypertrophy and higher blood pressure [34–38].

Abdominal adiposity was most strongly associated with metabolic syndrome in Dutch women. Although the prevalence of abdominal obesity is highest in Indonesian women, the relative contribution of abdominal obesity to metabolic syndrome is higher in Dutch women. The highest odds ratio of metabolic syndrome per SD of waist circumference in Dutch women may due to the accumulation of the highest risks of hypertriglyceridemia and low HDL-cholesterol in Dutch women, as well as the higher SD of waist circumference in the Dutch compared with the Indonesian.

Strengths of the present study are the large populations from the two countries, and the present study being the first to investigate the ethnic difference in the associations of body fat distribution with cardiometabolic complications. However, this study has several limitations. First, we realize that the existing ethnic-specific cut-offs, which were different for Asian and Caucasian population, influence the estimated prevalence of metabolic syndrome. Nevertheless, the patterns of associations between the body fat measures and metabolic syndrome were generally similar between the populations. Second, a possible underestimation of the prevalence of dyslipidemia in the Indonesian population due to the unavailability of the data on the use of lipid-lowering agents. However, we do not expect a large underestimation as statins, being the solely available lipid-lowering agent in the primary healthcare facilities, was not routinely prescribed [39]. Third, a possible overestimation of hyperglycemia and diabetes in the Indonesian population, as the glucose measures were estimated with a capillary blood test (Accu-Chek Performa, Roche Diagnostics GmbH, Mannheim, Germany) instead of a plasma sample, for logistic reasons [40]. Nevertheless, three studies have shown that the absolute relative difference in glucose concentrations between capillary blood testing and plasma was low (4.5%), resulting in no effect on diabetes diagnosis [41–43].

Several relevant clinical implications can be synthesized from this study. First, our results suggest a potential problem of undiagnosed diabetes. This was shown by the discrepancy of self-reported diabetes and the actual diagnosis of diabetes based on plasma glucose concentrations, particularly in the Indonesian population. The high prevalence of hyperglycemia in the populations also poses a further impending diabetes threat [44]. Second, the disparities between the prevalence of hypertension and the proportion of the population treated with anti-hypertensive medications may indicate inadequate treatment and/or uncontrolled hypertension. Third, extra attention may need to be given to those who are more prone to metabolic syndrome (e.g., to women in Indonesia, to men in the Netherlands). Finally, the distribution and contribution of metabolic syndrome components that were specific to each population may help doctors to plan population-specific healthcare strategies that are timely and relevant for their communities. Since 90% and 81% of the Indonesian and the Dutch population had at least one cardio-metabolic abnormality, routine screenings may also be warranted in younger adults (< 45 years) to enable the prevention of metabolic syndrome.

## Conclusion

Metabolic syndrome is sex- and population-specific. More Indonesian women than men have metabolic syndrome, whereas the opposite is true for the Dutch population. Although hypertension was shown to be the most prominent component in the Indonesian and Dutch population, the contributions of metabolic syndrome components differed between the two populations. In both populations, abdominal adiposity was more strongly associated with metabolic syndrome than overall adiposity. Population-specific healthcare strategies may be highly advantageous to prevent metabolic syndrome in the multi-ethnic global population.

## Supplementary information

**Additional file 1.** Additional tables.

## Data Availability

The datasets analyzed during this study are available on reasonable request from the NEO Study Board (https://www.lumc.nl/org/neo-studie/), and the Ministry of Health, Republic of Indonesia (http://labdata.litbang.depkes.go.id/).
