# Supplementary material for: The prevalence of metabolic syndrome and its association with body fat distribution in middle-aged individuals from Indonesia and the Netherlands: a cross-sectional analysis of two population-based studies
Source: Diabetol Metab Syndr. 2020 Jan 7;12:2. doi: 10.1186/s13098-019-0503-1 (PMC6947940; doi:10.1186/s13098-019-0503-1)
Supplement: Supplementary file 1 — Additional file 1. Additional tables. [file 13098_2019_503_MOESM1_ESM.docx]

**[Additional file 1: Table S1] The Contributions of Metabolic Syndrome Components in the Indonesian and Dutch Population**

|  | **Cardio-metabolic Abnormalities** | **Number of**  **Components** | **%** | | | |
| --- | --- | --- | --- | --- | --- | --- |
|  |  |  | **Indonesian** | | **Dutch** | |
| **1** | AO-HG-HTG-HDL-HT | 5 | 5.0 | | 5.6 | |
| **2** | AO-HG-HTG-HDL | 4 | 1.4 | 12.4 | 1.1 | 9.9 |
| **3** | AO-HTG-HDL-HT |  | 2.6 |  | 3.0 |  |
| **4** | AO-HG-HTG-HT |  | 1.9 |  | 2.3 |  |
| **5** | AO-HG-HDL-HT |  | 3.9 |  | 1.1 |  |
| **6** | HG-HTG-HDL-HT |  | 2.6 |  | 2.4 |  |
| **7** | AO-HG-HT | 3 | 6.4 | 21.6 | 5.0 | 14.1 |
| **8** | AO-HG-HTG |  | 0.4 |  | 0.4 |  |
| **9** | HG-HTG-HDL |  | 1.7 |  | 0.4 |  |
| **10** | HTG-HDL-HT |  | 1.5 |  | 2.9 |  |
| **11** | AO-HDL-HT |  | 3.0 |  | 1.5 |  |
| **12** | HG-HDL-HT |  | 2.8 |  | 0.2 |  |
| **13** | HG-HTG-HT |  | 2.3 |  | 1.0 |  |
| **14** | AO-HTG-HDL |  | 0.8 |  | 0.8 |  |
| **15** | AO-HG-HDL |  | 1.7 |  | 0.3 |  |
| **16** | AO-HTG-HT |  | 1.0 |  | 1.6 |  |
| **17** | AO-HG | 2 | 2.3 | 28.4 | 2.0 | 21.1 |
| **18** | HG-HT |  | 8.3 |  | 5.7 |  |
| **19** | HG-HTG |  | 1.2 |  | 0.4 |  |
| **20** | AO-HTG |  | 0.3 |  | 0.6 |  |
| **21** | HTG-HDL |  | 1.4 |  | 0.8 |  |
| **22** | HG-HDL |  | 2.6 |  | 0.2 |  |
| **23** | HDL-HT |  | 2.7 |  | 1.0 |  |
| **24** | HTG-HT |  | 1.7 |  | 1.6 |  |
| **25** | AO-HT |  | 6.1 |  | 8.0 |  |
| **26** | AO-HDL |  | 1.8 |  | 0.8 |  |
| **27** | AO | 1 | 2.8 | 22.8 | 6.2 | 30.1 |
| **28** | HG |  | 6.4 |  | 3.1 |  |
| **29** | HTG |  | 1.2 |  | 0.6 |  |
| **30** | HDL |  | 3.0 |  | 1.2 |  |
| **31** | HT |  | 9.4 |  | 19.0 |  |
| **32** | No Components | 0 | 9.6 | | 19.4 | |

Data were presented in %. Results were based on analyses weighted towards geographical density across 33 provinces (in the Indonesian population) and towards a normal BMI distribution (in the Dutch population). AO: Abdominal Obesity; HG: Hyperglycemia; HTG: Hypertriglyceridemia; HDL: Low HDL-Cholesterol; HT: Hypertension.

**[Additional file 1: Table S2] The Contributions of Components in Individuals with Metabolic Syndrome**

|  | **Indonesian** | | | **Dutch** | | |
| --- | --- | --- | --- | --- | --- | --- |
|  | Total | Men | Women | Total | Men | Women |
| Hyperglycaemia | 72.1 (0.8) | 79.1 (1.2) | 69.4 (1.0) | 66.1 (1.2) | 68.2 (1.6) | 63.5 (1.9) |
| Hypertension | 80.6 (0.7) | 79.1 (1.2) | 81.2 (0.9) | 90.0 (0.7) | 92.4 (0.8) | 87.1 (1.2) |
| Hypertriglyceridemia | 51.9 (0.9) | 73.6 (1.3) | 43.4 (0.9) | 72.4 (1.0) | 79.5 (1.0) | 63.8 (1.7) |
| Low HDL-Cholesterol | 76.0 (0.7) | 73.1 (1.3) | 77.1 (0.8) | 67.3 (1.1) | 67.7 (1.4) | 66.9 (1.7) |
| Abdominal Obesity | 70.9 (0.8) | 41.1 (1.4) | 82.5 (0.7) | 76.2 (1.4) | 65.7 (1.9) | 88.8 (1.8) |

Data were presented in % (SE). Results were based on analyses weighted towards geographical density across 33 provinces (in the Indonesian population) and towards a normal BMI distribution (in the Dutch population). This subpopulation analyses were conducted within metabolic syndrome patients only.

**[Additional file 1: Table S3] The Associations of Overall and Abdominal Adiposity with Components of Metabolic Syndrome**

| **BMI** | **Indonesian (SD = 4.4 kg/m^2^)** | | | | **Dutch (SD = 4.4 kg/m^2^)** | | | |
| --- | --- | --- | --- | --- | --- | --- | --- | --- |
|  | **Men** | | **Women** | | **Men** | | **Women** | |
|  | **Crude β** | **Adjusted β** | **Crude β** | **Adjusted β** | **Crude β** | **Adjusted β** | **Crude β** | **Adjusted β** |
| Systolic Blood Pressure (mmHg) | 5.3 (4.1, 6.5) | 3.4 (2.0, 4.9) | 3.8 (3.0, 4.6) | 3.4 (2.2, 4.5) | 3.7 (2.8, 4.5) | 4.5 (2.4, 6.6) | 2.0 (1.3, 2.7) | 2.2 (0.6, 3.7) |
| Diastolic Blood Pressure (mmHg) | 3.7 (3.0, 4.4) | 2.2 (1.4, 3.0) | 3.4 (2.9, 3.8) | 2.4 (1.7, 3.0) | 2.5 (1.9, 3.1) | 3.2 (1.8, 4.6) | 2.0 (1.6, 2.3) | 2.2 (1.3, 3.2) |
| Serum Triglyceride (mmol/L) | 0.3 (0.2, 0.3) | 0.1 (0.0, 0.2) | 0.1 (0.1, 0.2) | 0.1 (0.0, 0.1) | 0.4 (0.3, 0.4) | 0.1 (0.0, 0.2) | 0.2 (0.2, 0.2) | -0.0 (-0.1, 0.0) |
| HDL Cholesterol (mmol/L) | -0.1 (-0.1, -0.0) | -0.0 (-0.1, -0.0) | -0.1 (-0.1, -0.0) | -0.0 (-0.1, -0.0) | -0.2 (-0.2, -0.1) | -0.1 (-0.1, -0.0) | -0.2 (-0.2, -0.2) | 0.0 (-0.0, 0.0) |
| Fasting Plasma Glucose (mmol/L) | 0.3 (0.2, 0.4) | 0.1 (-0.0, 0.2) | 0.1 (0.0, 0.1) | -0.0 (-0.1, 0.1) | 0.4 (0.3, 0.4) | 0.1 (-0.0, 0.2) | 0.3 (0.3, 0.3) | 0.1 (0.0, 0.1) |

| **Waist Circumference** | **Indonesian (SD = 11.6 cm)** | | | | **Dutch (SD = 13.4 cm)** | | | |
| --- | --- | --- | --- | --- | --- | --- | --- | --- |
|  | **Men** | | **Women** | | **Men** | | **Women** | |
|  | **Crude β** | **Adjusted β** | **Crude β** | **Adjusted β** | **Crude β** | **Adjusted β** | **Crude β** | **Adjusted β** |
| Systolic Blood Pressure (mmHg) | 4.7 (3.8, 5.7) | 2.4 (1.1, 3.7) | 4.0 (3.0, 4.7) | 1.8 (0.6, 3.0) | 3.1 (2.2, 4.0) | -1.0 (-3.1, 1.1) | 1.9 (1.2, 2.7) | -0.6 (-2.3, 1.1) |
| Diastolic Blood Pressure (mmHg) | 3.2 (2.6, 3.8) | 1.5 (0.7, 2.2) | 3.2 (2.9, 3.6) | 1.5 (0.9, 2.1) | 2.0 (1.4, 2.6) | -0.9 (-2.2, 0.5) | 1.7 (1.3, 2.2) | -0.3 (-1.4, 0.7) |
| Serum Triglyceride (mmol/L) | 0.3 (0.2, 0.3) | 0.2 (0.1, 0.3) | 0.2 (0.1, 0.2) | 0.1 (0.1, 0.2) | 0.3 (0.3, 0.4) | 0.2 (0.1, 0.3) | 0.3 (0.2, 0.3) | 0.2 (0.2, 0.3) |
| HDL Cholesterol (mmol/L) | -0.1 (-0.1, -0.0) | -0.0 (-0.1, -0.0) | -0.1 (-0.1, -0.0) | -0.0 (-0.1, -0.0) | -0.2 (-0.2, -0.1) | -0.1 (-0.1, -0.0) | -0.2 (-0.2, -0.2) | -0.2 (-0.2, -0.1) |
| Fasting Plasma Glucose (mmol/L) | 0.2 (0.2, 0.3) | 0.1 (-0.0, 0.2) | 0.2 (0.1, 0.2) | 0.2 (0.1, 0.2) | 0.3 (0.3, 0.4) | 0.0 (-0.1, 0.1) | 0.3 (0.3, 0.3) | 0.1 (0.0, 0.1) |

**Regression coefficients (β) of metabolic syndrome components per 1 SD of BMI waist circumference**. Data were presented as β (95%CI). Interpretation: 1 SD of waist circumference in Indonesian men is associated with 2.4 mmHg higher systolic blood pressure after adjustment. Multivariate were adjusted for age, education, smoking behavior, physical activity, pre-existing CVD, Stroke, and Diabetes. In the Indonesian population: additionally adjusted for urban/rural, and socioeconomic status. In the Dutch population: additionally adjusted for alcohol consumption, menopausal status, and hormone use. BMI and waist circumference were mutually adjusted.
